# History of a Case of Ischuria Successfully Treated with the Cold Affusion

**Published:** 1828-07

**Authors:** Lewis Campbell

**Affiliations:** Moulton, Alabama


WESTERN JOURNAL

OF THE

### MEDICAL & PHYSICAL SCIENCES.

JULY, 1828.

### ESSAYS AND CASES.

—

ART. I.—*History of a case of* ISCHURIA, *successfully treated by the cold affusion.* *By* DR. LEWIS CAMPBELL, *of Moulton, Alabama.*

On Sunday, the 29th of May, 1827, I was called to H. M. W—, Esq., aged thirty four. He informed me, that he was taken, on the preceding Wednesday, with dysenteric symptoms. Being from home on business, he continued to ride a short distance, every day, till Saturday, when he was confined. In the morning before I saw him, however, he had rode 15 miles in a gig. When I arrived, he complained of intense pain in the region of the rectum and bladder, with continued and distressing tenesmus, and an entire suppression of urine, which had existed for forty eight hours. His skin was dry and warm, pulse frequent and tense, stools scanty and whey coloured, covered with a black flaky matter, stomach irritable.

He was placed in a tub of tepid water and had a vein opened, which bled freely; but although kept in the bath for some time, no effect was produced on the strangury. I proposed the use of the catheter; at the same time expressing my reluctance to attempt its introduction, as it would be necessarily attended with pain, and, perhaps, considerable difficulty, from the inflamed and irritable state of the parts.

To this operation, Mr. W. was reluctant to submit, while there was a hope of relief from any other means. He was again immersed in tepid water, and remained some time, but without effect.

In this trying situation I reflected, that cold, applied to the skin, has a tendency to produce a flow of urine. But a cold bath, in a case of inflammatory dysentery, would, as far as I knew, be an unprecedented practice. I did not, however, apprehend any danger from a current of cold water to the pubic region, whilst he was placed in tepid water to the nates. I accordingly took a half gallon pitcher of cold spring water, and poured it, in a continued stream, on the region of the bladder and pubis, while he sat in the tepid bath. Before I had emptied the third pitcher, the urine broke forth, in a full and free stream, greatly to my satisfaction, and to the inexpressible relief of the patient.

I then directed ʒj. of Balsam Copavi and 10 grains of Pulv. Doveri, with a dose of Calomel, to be taken in the course of the evening.

30th, at 8 o'clok, A. M. I found that the patient had neglected to take the Calomel, and had spent a restless night. His calls to the stool were frequent and painful. The dejections had the same appearance as yesterday. He complained of pain in the course of the urethra; had had no evacuation of urine. Gave the dose of calomel; and the same expedient which succeeded so well last evening, was resorted to with equal success. The stream of cold water had not been applied thirty seconds, when the urine burst forth; the bladder emptying itself completely. At 7 o'clock, P. M., the calomel had operated freely. The stools were dark and consistent, with great pain, and a total loss of the power of the sphincter ani, the contents of the bowels passing off involuntarily. Laudanum and Dover's powder were both taken during the night.

31st, 8 oclock, A. M. Dejections were copious and dark coloured, pulse frequent. Six grains of calomel and thirty drops of laudanum were ordered. The stools improved in their appearance during the day. At 6 o'clock, P. M., Griping and tenesmus, with distressing pain in the region of the bladder; had not made water; pulse excited; skin dry. Drew twenty four ounces of blood, and used the water as before, with the same success.

June 1st, 6 o'clock, A. M. Directed 5 grains calomel and 15 grains pulv. Doveri. Eased the tenesmus, and occasioned a perspirable state of the surface. The alvine evacuations frequent and consistent; of a lighter colour than formerly; urine evacuated by the application of the water as before. At 3 o'clock, P. M., being called to the country for the night, I directed twenty grains pulv. Doveri, and a drachm of bals. Copaiva to be taken in the course of the night. This quantity of balsam had indeed been given every evening.

June 2d, 10 o'clock, A. M. Had not urinated since the morning of the preceding day. Extreme pain in the rectum and bladder. Omitted the powders last night. Gave the Dover's powder with five grains of calomel. Evacuation of urine effected as formerly, but with more difficulty and a longer continuance in the bath. Bowels were freely opened by an enema of warm water. From this time his convalescence was speedy and uninterrupted.

*Moulton, Alabama, May* 19, 1828.

